# Deltamethrin Selection Drives Transcriptomic Changes in Detoxification, Immune, and Cuticle Genes in *Aedes aegypti*

**DOI:** 10.3390/tropicalmed10060171

**Published:** 2025-06-17

**Authors:** Yamili Contreras-Perera, Lucy Mackenzie-Impoinvil, Dieunel Derilus, Audrey Lenhart, Iram P. Rodriguez-Sanchez, Pablo Manrique-Saide, Adriana E. Flores

**Affiliations:** 1 Facultad de Ciencias Biologicas, Universidad Autonoma de Nuevo Leon, Av. Universidad s/n, Cd. Universitaria, San Nicolas de los Garza 66451, Mexico; yamjaz_85@hotmail.com (Y.C.-P.); iram.rodriguezsa@uanl.edu.mx (I.P.R.-S.); 2 Laboratorio para el Control Biológico de Aedes aegypti (LCB-UADY), Unidad Colaborativa para Bioensayos Entomologicos, Campus de Ciencias Biologicas y Agropecuarias, Universidad Autonoma de Yucatan, Carretera Merida-Xmatkuil Km. 15.5, Merida 97315, Mexico; msaide@correo.uady.mx; 3 Entomology Branch, Division of Parasitic Diseases and Malaria, National Center for Emerging and Zoonotic Infectious Diseases, Centers for Disease Control and Prevention, 1600 Clifton Rd, Atlanta, GA 30329, USA; qlk5@cdc.gov (D.D.); ajl8@cdc.gov (A.L.)

**Keywords:** *Aedes aegypti*, deltamethrin selection, RNA-seq, cytochrome P450, peritrophic matrix, cuticular proteins, immune-related genes

## Abstract

The rapid global expansion of *Aedes aegypti*-borne diseases such as dengue, chikungunya, and Zika has positioned this mosquito as a key target for vector control programs. These programs rely heavily on insecticide use, leading to the widespread emergence of insecticide resistance. Understanding the molecular basis of resistance is essential for developing effective management strategies. In this study, we employed a whole-transcriptome (RNA-seq) approach to analyze gene expression in three *Ae. aegypti* populations from Mexico that underwent four generations of laboratory selection with deltamethrin. Several cytochrome P450 genes (CYP6AG4, CYP6M5, CYP307A1) and a chitin-binding peritrophin-like gene (Ae-Aper50) were significantly overexpressed following selection, supporting roles for both detoxification and midgut protection. We also observed a consistent downregulation of cuticular protein genes in deltamethrin-selected groups relative to the baseline populations, suggesting their involvement in baseline tolerance rather than induced resistance. Additionally, the overexpression of immune- and stress-related genes, including the RNA helicase MOV-10, indicates that insecticide selection may trigger broader physiological responses. These findings highlight complex, multi-pathway transcriptomic changes associated with resistance development in *Ae. aegypti*.

## 1. Introduction

Reducing vector-borne diseases (VBDs) is one of the most important public health challenges globally. *Aedes aegypti* (L.) is considered the primary vector of the viruses that cause dengue (DEN), chikungunya (CHIK), and Zika (ZIK), collectively known as *Aedes*-transmitted diseases (ATDs) [1]. These diseases are increasingly recognized as global public health threats [2,3,4]., with active circulation reported throughout Mexico [5].

The main strategy for mitigating these threats lies in the control of *Ae. aegypti* mosquitoes. Public health efforts have thus been directed toward a dual-pronged approach: minimizing potential mosquito breeding grounds and implementing chemical control measures such as larvicides and adulticides [6]. Many of these chemical vector control interventions rely on the use of neurotoxic insecticides, which are commonly employed against *Ae. aegypti* [7,8].

Pyrethroids are the most widely used insecticides around the world due to their low toxicity to mammals, including humans [9], and their high selectivity in insects [10]. In Mexico, pyrethroids have been a central component of *Ae. aegypti* control efforts since the withdrawal of DDT in the late 1990s, and their extensive use has been well documented as part of national vector control programs [11]. However, their widespread and prolonged use for over 20 years has led to the emergence of highly resistant *Ae. aegypti* populations, which have been documented in several regions of the country [11,12,13,14,15,16].

Several mechanisms have been described by which mosquitoes develop resistance to insecticides. Among these are the knockdown resistance (kdr) point mutations on the voltage-gated Na+ channel (VGSC), which confer resistance to both pyrethroids and DDT. The VGSC in *Ae. aegypti* is essential for the initiation and propagation of the action potential in neurons and other excitable cells. It is a transmembrane protein present in the neuronal axons, composed of four homologous domains (I–IV), each with six hydrophobic segments (S1–S6) [17]. To date, at least thirteen kdr mutations have been reported in *Ae. aegypti*, of which five have been functionally confirmed to confer resistance to pyrethroids. These mutations include S989P (link IIS5-S6), I1011M, V1016G/I (IIS6), F1534C (IIIS6), and V410L (IS6) [18,19]. Three of these (F1534C, V1016I, and V410L) have been detected in pyrethroid-resistant *Ae. aegypti* populations in Mexico [20].

Another important mechanism of resistance is the enhanced detoxification of insecticides driven by elevated metabolic activity. This is typically associated with the increased activity of three major enzyme families: esterases, mixed-function oxidases, and glutathione S-transferases [21,22]. Previous research conducted in Mexico has documented detoxification enzyme activity in pyrethroid-resistant *Ae. aegypti* populations [12,14,15,23,24,25,26,27].

Kdr mutations and increased metabolic activity have been the most commonly reported mechanisms of pyrethroid resistance [21]. However, modifications in the insect cuticle may also contribute to pyrethroid tolerance [28]. The cuticle is a multifunctional structure that provides physical protection and structural support and serves as a barrier against desiccation, pathogens, and chemical agents. In mosquitoes and other arthropods, the cuticle plays a critical role in environmental adaptation and insecticide interaction [29]. The structure and regulation of cuticle proteins are key determinants of resistance to insecticides, as they influence the composition and permeability of the cuticle, thereby limiting the penetration of toxic compounds [30]. Electron microscopy studies have linked cuticle thickness to permethrin tolerance in the malaria vector *Anopheles funestus*, showing significantly thicker cuticles in resistant individuals compared to susceptible ones [31]. Additionally, RNA interference assays have identified a new resistance mechanism in the malaria vector *Anopheles gambiae*, involving a sensory appendage protein (SAP2) located on the legs, which has demonstrated an affinity for binding pyrethroids [32]. Sensory appendage proteins (SAPs), a type of small, soluble protein found only in arthropods, belong to the chemosensory protein (CSP) family [33]. These proteins assist in transporting hydrophobic compounds through the sensillum lymph [34]. SAP2 has been implicated as a key factor in pyrethroid resistance. Its overexpression in resistant populations, increased induction upon insecticide exposure, and ability to bind pyrethroids suggest that SAP2 may confer resistance by sequestering the insecticide at the point of contact, thereby preventing its neurotoxic action or facilitating subsequent detoxification [32].

Furthermore, changes in the expression of regulatory genes that code for other processes such as metabolism, detoxification and excretion [21,35], replication, transcription, structural function, synaptic function [36], cellular respiration, endopeptidase activity, extracellular activity, and chitin metabolism could also play an important role in pyrethroid resistance [37,38]. The increased use of whole-transcriptome approaches to study gene expression patterns sheds light on how genes regulating these processes are associated with insecticide resistance in important disease vectors.

This study aimed to identify the gene transcription profiles induced by four generations of deltamethrin selection in natural populations of *Ae. aegypti* from Mexico

## 2. Materials and Methods

### 2.1. Aedes aegypti Populations

Eggs and larvae of Ae. aegypti were collected from three distinct localities in Mexico. The first site was Jose Cardel in Veracruz state (JC), located in the Gulf of Mexico region (19°22′15″ N, 96°22′35″ W). This site is characterized by a warm, humid climate with year-round rainfall and intense urban activity, providing ideal breeding conditions for Ae. aegypti. The second site was the Manzana 115 neighborhood in the city of Merida (MER), Yucatan state (20°56′42″ N, 89°38′36″ W), situated in the Yucatan Peninsula in southern Mexico. This region has a tropical savanna climate with distinct dry and rainy seasons, and Ae. aegypti is well established due to the widespread use of domestic water storage containers. The third site was the town of Hunucma (HUN) (21°00′58″ N, 89°52′38″ W), also in the Yucatan Peninsula, known for its peri-urban and rural landscape with frequent use of wells and small water containers. Immature stages (larvae and pupae) were collected from common, clean, stored, or stagnant water containers, such as buckets, barrels, and discarded items, ensuring that at least 10 different sampling sites were included in each locality to reflect local habitat variability.

These samples were transported to the insectary of the Medical Entomology Laboratory at the Universidad Autonoma de Nuevo Leon (UANL), where they were maintained at 28 ± 1 °C, 70–80% relative humidity, and a 12:12 h (light: dark) photoperiod until adult emergence. This generation was designated as F_S0_ (non-selected generation). As a susceptible reference strain, the New Orleans strain (NOr) of *Ae. aegypti*, originally obtained from the CDC (Atlanta, GA, USA) and maintained at UANL since 2002, was used for comparative purposes.

### 2.2. Bioassays and Deltamethrin Selection

Technical grade deltamethrin (99.5%) (Chemservice, West Chester, PA, USA) was used for both bioassays and selection procedures. Bottle bioassays were conducted according to a standardized protocol [39], using 3–to 5-day-old mosquitoes maintained on 10% sucrose. For each population, 20–25 nonblood-fed females were tested per concentration, across 7 to 10 different concentrations that produced between 9 and 90% mortality. Each concentration was tested in triplicate, and untreated control bottles were included in every assay. Mortality was recorded 24 h after 1 h of exposure and analyzed using logistic regression to estimate the LC_50_ (concentration causing 50% mortality) with QCal https://sourceforge.net/projects/irmaproj/files/Qcal/ (accessed on 18 January 2024) [40]. Correction of mortality was not necessary, as no mortality was observed in the controls.

For the selection procedure with deltamethrin, 650–1000 mosquitoes per generation, including both males and females at a 1:4 ratio, were exposed for 1 h to the LC_50_ determined for the previous generation. Approximately 180–350 survivors (males and females) were transferred to entomological cages for breeding and progeny production. The LC_50_ was recalculated in females for each subsequent generation and used in the following selection cycle. This process was repeated until the F_S4_ generation.

### 2.3. RNA and Library Preparation

Four-to-five-day-old adult nonblood-fed female mosquitoes were killed by freezing and stored at −80 °C. For each of the three locations, three biological replicates consisting of pools of five female mosquitoes for each group were collected. This included the Hunucma non-selected baseline generation (HUNF_S0_) and insecticide-selected fourth generation (HUNF_S4_), Merida non-selected baseline generation (MERF_S0_) and insecticide-selected fourth generation (MERF_S4_), Jose Cardel non-selected baseline generation (JCF_S0_) and insecticide-selected fourth generation (JCF_S4_) as well as from the New Orleans strain (NOr). RNA was extracted from each pool using the Applied Biosystems^®^ Arcturus^®^ PicoPure^®^ RNA isolation kit (Arcturus, biological Biosystems, San Diego, CA, USA) by homogenizing the mosquitoes in the provided extraction buffer and following the protocol for the remaining steps according to the manufacturer’s instructions. The RNA concentration, quality, and integrity were analyzed using an Agilent 4200 TapeStation bioanalyzer (Agilent Technologies, Palo Alto, CA, USA) using the RNA ScreenTape kit. The DNA remnants were removed with the Baseline-ZERO™ DNase removal kit (Epicenter, Illumina, San Diego, CA, USA), and ribosomal RNA was removed with the Ribo-Zero ™ rRNA removal kit (Human/Mouse/Rat) (Epicenter, Illumina). Although this kit is not specifically designed for mosquitoes, it has been successfully applied in previous transcriptomic studies of *Ae. aegypti* [41], and rRNA removal was confirmed based on RNA quality profiles obtained using the Agilent 4200 TapeStation. The quantity and size distributions of the libraries were assessed using the Agilent DNA ScreenTape assay (Agilent, Santa Clara, CA, USA).

Library preparation was carried out using the ScriptSeq™ v2 RNA-seq Library Preparation Kit (Epicenter, Illumina) according to the manufacturer’s instructions, and each library was barcoded. Equal amounts of each library were then pooled and sequenced at 2 × 125 bp paired-end on an Illumina HiSeq 2500 sequencer (Illumina, San Diego, CA, USA) using the v2 chemistry at the biotechnology core facility at the CDC in Atlanta, GA, USA.

### 2.4. Differential Gene Expression Analysis

The quality of the sequences obtained was assessed using FastQC v0.11.5 [42]. To remove adapters and low-quality reads, the sequencing reads were trimmed and filtered with Fastp software v0.20.1 by using the following non-default parameters: minimum length required set to 25 (-l 25), the polyX were trimmed in 3′ ends of each sequence (-x), one low-quality base was trimmed at the tail of both forward and reverse of each paired-end reads option (--trim_tail1 1 and --trim_tail1 2 1) [43]. The filtered read pairs (R1/R2) were aligned to the reference genome of *Ae. aegypti* (version = AaegL5, VectorBase release = 51) by using ‘subjunc’, which is part of the Subread aligner package, v2.0.1 [44]. The alignment was performed using the following non-default parameters: Maximum mismatches allowed = 5 (-M) and Maximum fragment/insert length = 1500 (-D). The low-quality alignments and the unmapped reads were filtered using Samtools, version 1.18 [45]. Finally, the alignments assigned to protein-coding genes were summarized and counted using ‘featurecount,’ part of the Subread aligner, v2.0.1 [44]. The featurecount analysis was carried out using the following non-default parameters: -p (count fragment instead of reads), -B (consider only fragments that have both ends successfully aligned), -C (avoid counting chimeric fragments) -Q = 10 (the minimum mapping quality score a read must satisfy to be counted). The complete bioinformatics pipeline with this list of command lines and software used for quality filtering, alignment, and feature counts is provided in File S1.

To identify gene expression patterns associated with deltamethrin selection, differential gene expression (DGE) analysis was performed for three main comparisons: F_S0_ vs. NOr, F_S4_ vs. F_S0_, and F_S4_ vs. NOr. The first comparison screened for the DGEs (JC, MER, and HUN) of the non-selected baseline generation (F_S0_) compared to the susceptible strain (NOr); the second and third comparisons identified genes whose differential expression was more likely driven by the insecticide selection. DGEs that overlapped the two last comparisons provided stronger evidence of their potential association with insecticide selection.

The differential gene expression analysis was carried out at the ‘gene’ level using the EdgeR package (v3.28.1) [43,46]. To explore the level of similarity in gene expression between all samples, double clustering analysis was performed for the tag count using principal component analysis (PCA) and hierarchical clustering using ggplot2 [47] and heatmap package [48], respectively. The bioinformatics pipeline for the DGE analysis of this dataset was previously described by Derilus et al. [41] and is provided in File S2. Briefly, the DGE analysis was performed on the feature count table as follows: (1) removal of lowly expressed genes (total count ≤ 50) for each pairwise comparison; (2) normalization of tag count results for sequencing depth and library size using the trimmed mean M-values (TMM) method; (3) estimation of the common, trended, and tagwise dispersions across all tags using *estimateDisp* function; (4) fitting of a negative binomial generalized log-linear model to the read counts for each gene using *glmFit* function; (5) conducting likelihood ratio tests for one or more coefficients in the linear model using *glmLRT* function; and (6) identifying genes that were significantly differentially expressed for each contrast with *decideTest* function, by adjusting the false discovery rate (FDR), using the Benjamini Hochberg (BH) procedure. Only with an absolute log2 fold change |log_2_FC| = 1 (|FC| = 2) and a false discovery rate (FDR)-adjusted *p* value ≤ 0.01 were considered significantly differentially expressed.

### 2.5. Gene Ontology Annotation and Functional Enrichment Analysis

To complement the VectorBase annotation, gene ontology (GO) and functional annotation of the AaegL5 gene set were performed locally using the Blast2GO command line (v1.4.4) [49] as described previously [41]. The annotations from Blast2GO and VectorBase annotation were then combined. The resulting annotated genes and their associated GO terms were used as the background reference set for functional GO enrichment analysis (GOEA) of the DGEs. GOEA was conducted using Goatools [50], using the default parameters. GO terms were considered significantly enriched if they had a false discovery rate (FDR)-adjusted *p*-value < 0.05. Non-coding genes and pseudogenes were excluded from the GO enrichment analysis due to the lack of comprehensive functional annotation in existing *Ae. aegypti* databases.

## 3. Results

### 3.1. Bioassays and Selection with Deltamethrin

The non-selected generation (F_S0_) from each population showed resistance to deltamethrin when compared to the susceptible New Orleans (NOr) strain. The resistance ratio (RR_50_) was highest in the MER population at 134, followed by JC at 41 and HUN at 6. After four generations of selection with deltamethrin, a further decrease in susceptibility was observed. The resistance ratio (RR_50_) of the F_s4_ generation compared to the NOr strain increased to 193 for MER, 93 for JC, and 33 for HUN. Additionally, when comparing the F_s4_ generation to its baseline (F_S0_), MER showed a 1.44-fold reduction in susceptibility, JC had a 1.37-fold reduction, and HUN exhibited the greatest change, with F_s4_ being 5.32 times less susceptible than F_S0_ (Table 1).

### 3.2. Sequencing, Alignment, and Read Quantification

High-Seq Illumina paired-end sequencing generated approximately 7.1–49.4 million paired-end reads (R1 + R2) per cDNA-library referred to as a sample. After quality control filtering, 7.0–48.4 million paired-end reads remained for each sample, which represents 97 to 98% of the total reads. 5.0 to 33 million (54 to 75%) of the quality filtered reads were uniquely mapped to the reference genome AaegL5 (Table S1). Uniquely mapped reads consisted of single-ended reads or paired-end sets that map to exactly one location in the reference genome. The remaining portion of the total filtered reads was mapped to multiple regions of the genome. The read quantification results indicated that 41 to 81% of the good-quality alignments were successfully assigned to annotated protein-coding genes (Table S1). The transcriptional signal was detected in 92% of the predicted gene set of *Ae. aegypti* (AaegL5_1), indicating a high transcriptome coverage from this RNA-seq experiment.

### 3.3. Data Exploration

**Figure 1:**
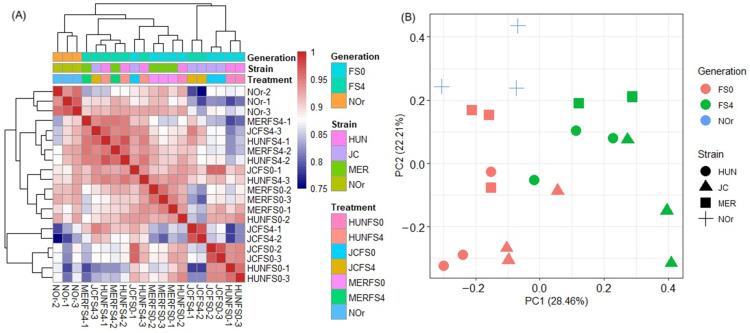
Clustering of the normalized RNA-seq data: (**A**) Hierarchical clustering heatmap of the sample-to-sample Pearson’s correlation of the normalized gene expression data assigned to each biological replicate; (**B**) principal component analysis (PCA) of multiple RNA-seq datasets.

To estimate the level of similarity in the cDNA libraries between treatments as well as biological replicates, a hierarchical clustering analysis of Pearson’s correlation of the normalized gene expression data was performed and visualized as a clustering heatmap (Figure 1A). Additionally, a principal component analysis (PCA) was conducted to explore the similarity of our samples as a measure of quality control. The PCA revealed that 28.46% and 22.21% of the total variation could be explained by PC1 and PC2, respectively. Each clustering analysis provided consistent results (Figure 1B). The RNA-seq libraries were grouped by generation but not by their origin or geographical location, suggesting that independent field populations are subjected to similar selection pressures in natural conditions. Likewise, the selected lines adapted to laboratory conditions and responded to the artificial selection pressure with deltamethrin in a similar manner. The NOr replicates clustered separately, consistent with its status as a laboratory-adapted reference strain that has been genetically conserved across many generations (Figure 1). Taken together, the biological replicates of the RNA-seq data for each treatment showed expected clustering patterns with no outliers, further confirming the sample quality.

### 3.4. Differentially Expressed Genes

Differential expression (DE) analysis of genes was carried out after quality control and removal of genes with low counts. The results include paired biological comparisons and the total number of differentially expressed (DE) genes for the experiments conducted on the JC, MER, and HUN populations. The comparisons include the baseline non-selected generation (F_S0_) versus the reference strain (NOr), the post-selection generation (F_S4_) versus the baseline (F_S0_), and the selected generation (F_S4_) versus the reference strain (NOr). Between 6672 and 10,188 genes were detected across biological comparisons. The F_S4_ vs. F_S0_ comparison highlighted the genes that responded to deltamethrin selection, with the highest number of DE genes observed in the JC population: 1235 (326 upregulated and 909 downregulated), followed by MER with 921 (311 upregulated and 610 downregulated) and HUN with 849 (181 upregulated and 668 downregulated) (Table 2). The full list of differentially expressed genes, including expression counts, fold change values, FDRs, and functional annotations, is provided in File S3.

**Figure 2:**
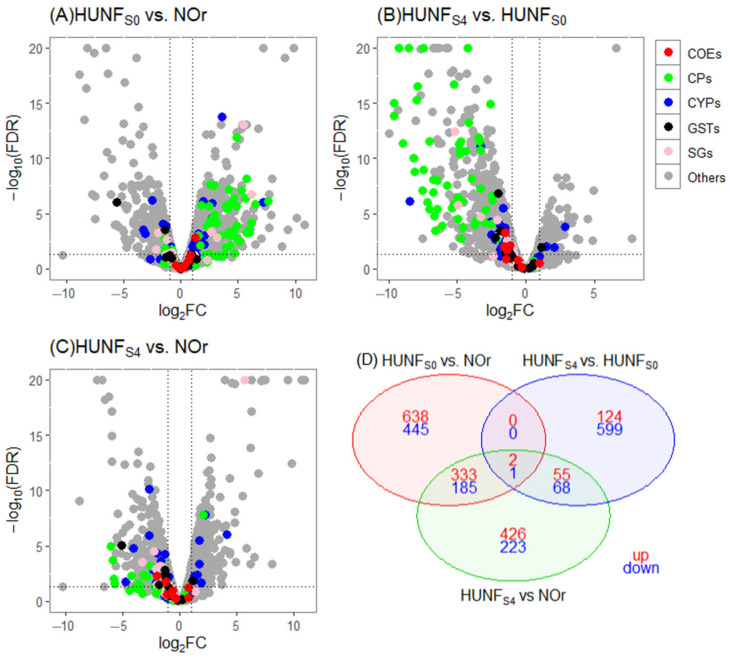
Volcano plots for gene expression for the HUN population experiment. (**A**) HUNF_S0_ vs. NOr, (**B**) HUNF_S4_ vs. HUNF_S0_, and (**C**) HUNF_S4_ vs. NOr. The *X*-axis shows log_2_ fold change (FC), where negative and positive values indicate down- and upregulation, respectively. The *Y*-axis shows −log_10_ of the adjusted *p*-value. Detoxification gene families are color-coded as follows: red for carboxylesterases (COEs), blue for cytochrome P450s (CYPs), and black for glutathione S-transferases (GSTs). Cuticular proteins (CPs) are shown in green, salivary gland proteins (SGs) are shown in pink, and genes with unknown functions are shown in gray. In each plot, genes overexpressed in the F_S4_ group appear on the right side (>0 on the *x*-axis). The vertical dotted lines indicate a log_2_FC threshold of ±1, and the horizontal dotted line represents the significance cutoff (FDR ≤ 0.01). (**D**) Venn diagram showing differentially expressed (DGE) genes shared among the comparisons F_S0_ vs. NOr, F_S4_ vs. F_S0_, and F_S4_ vs. NOr.

For the HUN experiment, a total of 1604 differential expressed (DE) genes were observed in the biological comparison HUNF_S0_ vs. NOr, with 973 (60.7%) overexpressed and 631 (39.3%) underexpressed. Among the overexpressed genes, 55 were related to cuticular proteins (CPs) and detoxifying enzymes: 39 (71%) correspond to CPs, 15 (27%) to cytochrome P450s (CYPs), and 1 (2%) to carboxylesterases (COEs) (Figure 2A).

The DE genes expressed after selection with deltamethrin in the comparison HUNF_S4_ vs. HUNF_S0_ totaled 849, with 181 (21%) overexpressed and 668 (79%) underexpressed. Among the overexpressed genes, three corresponded to CYPs: CYP6AG4 (log_2_FC = 2.87), CYP307A1 (log_2_FC = 2.06) and CYP6M5 (log_2_FC = 1.52) (Figure 2B; Table 3).

A biological comparison of the selected line versus the reference strain (HUNF_S4_ vs. NOr) revealed a total of 1293 DE genes, with 816 (63%) overexpressed and 477 (37%) underexpressed. Among the overexpressed genes, 8 corresponded to CYPs and 1 to CPs (Figure 2C). Overall, most differentially expressed cuticular-related proteins were upregulated in F_S0_ compared to NOr but downregulated in F_S4_ relative to both F_S0_ and NOr.

Finally, three DE genes were shared across the biological comparisons: HUNF_S0_ vs. NOr, HUNF_4S_ vs. HUNF_S0_, and HUNF_S4_ vs. NOr (2, overexpressed and 1, underexpressed). The overexpressed gene AAEL005507 (inhibitory POU isoform X4, log_2_FC = 1.25) is a transcription factor with a molecular function in DNA molding, and AAEL010712 (low-density lipoprotein receptor-related 4 isoform X1, log_2_FC = 1.02) encodes for LDL group lipoproteins (Figure 2D).

**Figure 3:**
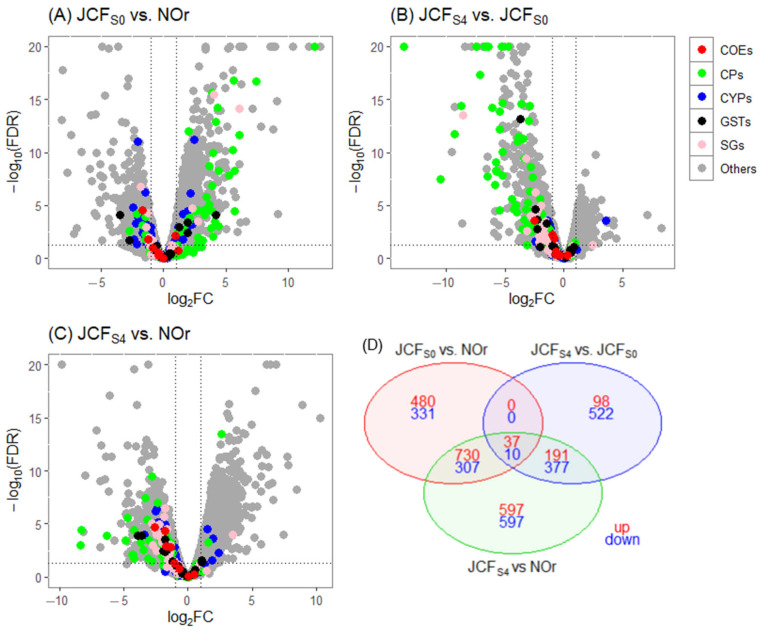
Volcano plots of gene expression in the JC population experiment. (**A**) JCF_S0_ vs. NOr, (**B**) JCF_S4_ vs. JCF_S0_, and (**C**) JCF_S4_ vs. NOr. The *X*-axis shows log_2_ fold change (FC), where negative and positive values indicate down- and upregulation, respectively. The *Y*-axis shows −log_10_ of the adjusted *p*-value. Detoxification gene families are color-coded as follows: red for carboxylesterases (COEs), blue for cytochrome P450s (CYPs), and black for glutathione S-transferases (GSTs). Cuticular proteins (CPs) are shown in green, salivary gland proteins (SGs) are shown in pink, and genes with unknown functions are shown in gray. In each plot, genes overexpressed in the F_S4_ group appear on the right side (>0 on the *X*-axis). The vertical dotted lines indicate a log_2_FC threshold of ±1, and the horizontal dotted line represents the significance cutoff (FDR ≤ 0.01). (**D**) Venn diagram showing differentially expressed (DGE) genes shared among the comparisons F_S0_ vs. NOr, F_S4_ vs. F_S0_, and F_S4_ vs. NOr.

In the Jose Cardel experiment, the DE genes were also grouped into the previously mentioned categories. For the biological comparison of JCF_S0_ vs. NOr, a total of 1895 DE genes were identified, with 1247 (66%) overexpressed and 648 (34%) underexpressed. Among the overexpressed genes, 35 corresponded to CPs, 9 to CYPs, and 4 to glutathione S-transferases (GSTs) (Figure 3A).

The DE genes identified after selection with deltamethrin in the comparison JCF_S4_ vs. JCF_S0_ included a total of 1235 genes, of which 326 (26%) were overexpressed and 909 (74%) were underexpressed. Among these, only one cytochrome P450 gene, CYP307A1 (log_2_FC = 3.53), was upregulated following deltamethrin selection (Figure 3B; Table 3).

The results of the biological comparison of the selected line versus the reference strain (JCFS4 vs. NOr) revealed a total of 2846 DE genes, with 1555 (55%) overexpressed and 1291 (45%) underexpressed. Among the overexpressed genes, four corresponded to CYPs and two to CPs (Figure 3C). Consistent with the HUN experiment, most differentially expressed cuticular-related proteins were upregulated in the F_S0_ compared to NOr but downregulated in F_S4_ relative to both F_S0_ and NOr.

The DE genes shared across the three comparisons (JCFS0 vs. NOr, JCFS4 vs. JCFS0, and JCFS4 vs. NOr) totaled 47, of which 37 (79%) were overexpressed and 10 (21%) were underexpressed (Figure 3D).

**Figure 4:**
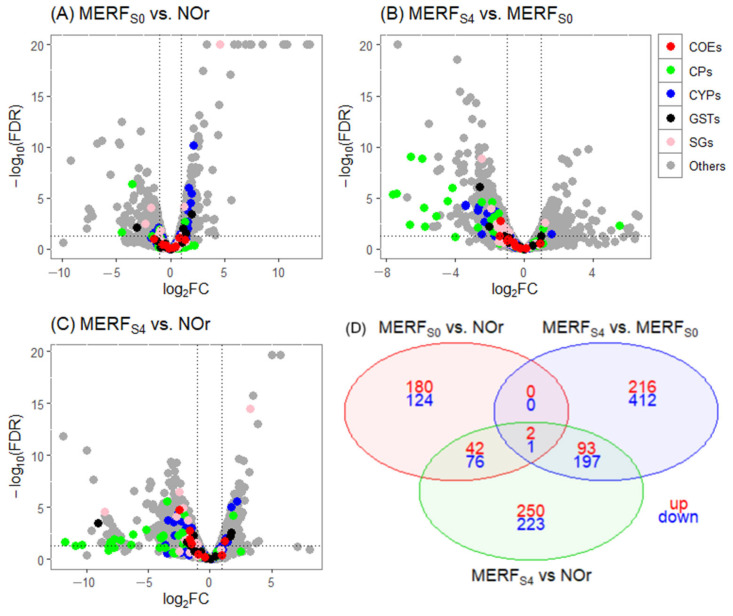
Volcano plots of gene expression in the MER population experiment. (**A**) MERF_S0_ vs. NOr, (**B**) MERF_S4_ vs. MERF_S0_, and (**C**) MERF_S4_ vs. NOr. The *X*-axis shows log_2_ fold change (FC), where negative and positive values indicate down- and upregulation, respectively. The *Y*-axis shows −log_10_ of the adjusted *p*-value. Detoxification gene families are color-coded as follows: red for carboxylesterases (COEs), blue for cytochrome P450s (CYPs), and black for glutathione S-transferases (GSTs). Cuticular proteins (CPs) are shown in green, salivary gland proteins (SGs) are shown in pink, and genes with unknown functions are shown in gray. In each plot, genes overexpressed in F_S4_ appear on the right side (>0 on the *X*-axis). The vertical dotted lines indicate a log_2_FC threshold of ±1, and the horizontal dotted line represents the significance cutoff (FDR ≤ 0.01). (**D**) Venn diagram showing differentially expressed (DGE) genes shared among the comparisons F_S0_ vs. NOr, F_S4_ vs. F_S0_, and F_S4_ vs. NOr.

The same comparisons were made for the experiment with the MER population. First, the biological comparison between MERF_S0_ vs. NOr revealed a total of 425 DE genes, with 224 (53%) overexpressed and 201 (47%) underexpressed. Among the overexpressed genes, one corresponded to CPs, seven to CYPs, and two to GSTs (Figure 4A).

In the biological comparison of MERF_S4_ vs. MERF_S0_ after selection with deltamethrin, a total of 921 DE genes were identified, with 311 (34%) overexpressed and 610 (66%) underexpressed. Among the overexpressed genes, only one was related to cuticular proteins (CUT): AAEL002467 (adult peritrophin 50, Ae-Aper50, FC = 5.52) (Figure 4B; Table 3).

The total number of DE genes from the biological comparison of the selected line versus the reference strain (MERF_S4_ vs. NOr) was 884, with 387 (44%) overexpressed and 497 (56%) underexpressed. Among the overexpressed genes, 5 correspond to CYPs, 1 to CPs, and 1 to GSTs (Figure 4C). Taken together, consistent with the HUN and JC experiments, most differentially expressed cuticular-related proteins were upregulated in the F_S0_ compared to NOr but downregulated in F_S4_ relative to both F_S0_ and NOr. This suggests that field- and lab-selected insecticide-resistant mosquitoes may exhibit different resistance mechanisms as evidenced by their transcriptomic profiles.

The DE genes shared across different comparisons (MERF_S0_ vs. NOr, MERF_S4_ vs. MERF_S0_, and MERF_S4_ vs. NOr) totaled 3, with 2 overexpressed and 1 underexpressed (Figure 4D).

**Figure 5:**
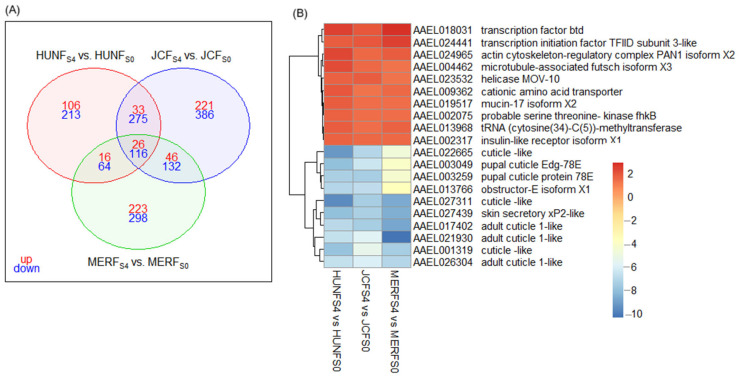
Shared differentially expressed genes (DGEs) in F_S4_ vs. F_S0_ comparisons across populations. (**A**) Venn diagram showing overlapping DEGs among F_S4_ vs. F_S0_ comparisons in HUN, JC, and MER populations. (**B**) Log_2_ fold change expression and functional annotation of the top 10 commonly upregulated and top 10 commonly downregulated DGEs shared across all F_S4_ groups. These shared genes represent a consistent transcriptomic response to deltamethrin selection when compared to their respective F_S0_ counterparts.

We identified 142 differentially expressed genes (26 up- and 116 downregulated), that were consistently shared among the three F_S4_ groups (HUN, JC, and MER) when compared to their respective F_S0_ counterpart (Figure 5). These overlapping DGEs represent a core transcriptomic response to deltamethrin selection across distinct populations. Upregulated core DGEs included two transcription factors (btd and TFIID subunit 3-like), an actin cytoskeleton-regulatory complex PAN1 isoform, Futsh protein, a cationic amino acid transporter, mucin-17, tRNA (cytosine34-C5)-methyltransferase, a probable serine/threonine kinase (fhkB), the helicase MOV-10, and an insulin-like receptor (lnR). In contrast, all top 10 downregulated core DGEs encoded cuticle protein homologs, suggesting that cuticle-based resistance mechanisms may have been negatively selected under deltamethrin pressure (Figure 5; File S4).

Enrichment analysis of gene ontology (GO) terms associated with these shared genes revealed consistent biological signatures across populations, as shown in Figure S1.

### 3.5. Gene Ontology Annotation and Enrichment Analysis

The AegL5 gene set contains over 19,804 predicted genes (14,718 protein-coding genes, 5086 non-protein-coding genes, and 382 pseudogenes). However, functional annotation is available for only 6319, and gene ontology (GO) annotation terms are assigned to 11,097 of the protein-coding genes. To enhance the interpretation of the data, the computational annotation of the AegL5 gene set was performed using Blast2GO (see Methods). This analysis assigned putative functional descriptions to 13,322 and GO terms to 11,680 of the protein-coding genes. All the annotation results from both VectorBase and Blast2GO are provided in File S5.

Gene ontology enrichment analysis (GOEA) was performed on the DE genes (up and downregulated, separately) from each F_S4_ vs. F_S0_ (3 in total). The significantly enriched GO terms, including biological process, molecular function, and cellular component categories, are reported in File S6. GO molecular function terms that were significantly enriched in at least two out of the three comparisons are summarized in Figure S1.

For the upregulated genes, GO molecular function terms associated with binding activities (ATP, nucleic acid, RNA, Adenyl ribonucleotide/nucleotide small molecule, organic cyclic compounds), transferase activity (methyl, one-carbon group), helicase, and ATP-dependent activities were significantly enriched, suggesting the potential role of these key molecular functions following insecticide selection. Of note, GO terms associated with methyltransferase activity were significantly enriched in the upregulated genes. Among these, tRNA (cytosine34-C5)-methyltransferase (AAEL013968) was consistently detected among the top 10 DE genes from all three F_S4_ vs. F_S0_ comparisons. This may suggest the involvement of the RNA methylation process in response to insecticide selection; however, direct evidence for DNA or RNA methylation in *Ae. aegypti* under deltamethrin exposure is currently lacking. Additionally, GO terms associated with helicase activity were enriched, with helicase MOV-10 protein (a stress-tolerant gene) among the top overexpressed genes in all three F_S4_ (Figure S1A). The GOEA of the downregulated genes revealed that GO terms associated with multiple oxidoreductases (heme group, NAD(P)H, CH-OH group), dehydrogenase (NADH), structural constituents (cuticle, chitin, and structural molecules), catalytic and transport activities were significantly enriched, indicating the potential downregulation of these molecular functions in deltamethrin-selected *Ae. aegypti* (Figure S1B).

## 4. Discussion

Several studies have demonstrated that the selection pressure exerted by insecticide exposure can lead to changes in gene expression in *Ae. aegypti* [36,51]. In general, exposure to any external agent (xenobiotic) can result in alterations in gene expression [52], which may contribute to the development of resistance. This resistance can manifest in various ways, such as structural modifications in the protein at the insecticide target site, increased biodegradation of the insecticide, and alteration of transportation or other functional groups [36,53]. Previous genomic studies in *Ae. aegypti* have revealed diverse detoxification genes, including members of the cytochrome P450, glutathione transferase (GST), and carboxy/cholinesterase families [54]. Among the cytochrome P450s genes, the CYP6 and CYP9 subfamilies are considered the main candidates involved in xenobiotic metabolism in this species [22,55]. Transcriptomic analyses conducted after permethrin selection indicated that at least ten cytochrome P450 genes were upregulated in multiple Mexican strains of *Ae. aegypti* [56]. Metabolic resistance mechanisms may also contribute to cross- and multi-resistance [57]. In addition, studies have examined the association between knockdown recovery and the frequency of VGSC mutations, as well as the expression of genes encoding cuticular proteins, detoxification enzymes, and other insecticide target sites [58,59].

This study identified transcriptomic responses to deltamethrin selection in three *Ae. aegypti* populations with distinct baseline resistance profiles. While F_S0_ populations already showed evidence of resistance linked to kdr mutations [15] and metabolic gene expression, F_S4_ mosquitoes exhibited additional expression changes involving detoxification enzymes, immune/stress-response factors, and structural components. These findings suggest that deltamethrin resistance in *Ae. aegypti* results from both inherited and selection-induced mechanisms, reflecting a multifactorial and population-specific adaptation to chemical pressure.

While the inclusion of three field-derived populations and a susceptible reference strain allowed for both intra- and inter-population comparisons of gene expression, we recognize that the number of populations analyzed may limit the generalizability of the findings. Additional populations from diverse ecological and genetic backgrounds would help to validate and expand the transcriptomic patterns observed here.

### 4.1. Baseline Resistance Profiles and Gene Expression in FS0 vs. NOr

The F_S0_ generations from MER, HUN, and JC exhibited notable differences in susceptibility to deltamethrin and gene expression profiles, reflecting their exposure to diverse selection pressures in their natural environments. These populations, originating from areas where multiple insecticides are commonly used [60], were likely exposed to selective agents other than deltamethrin, which may have contributed to their resistance profiles. The F_S0_ population from MER exhibited the highest resistance ratio (RR_50_ = 134) compared to the susceptible NOr strain, indicating a high level of resistance to deltamethrin. JC also showed high resistance (RR_50_ = 41), while HUN had the lowest resistance ratio (RR_50_ = 6.25). These differences in baseline resistance may be partly explained by the frequency of the tri-locus kdr genotype LL_410_/II_1016_/CC_1534_ previously reported for these populations [15]. MERF_S0_ exhibited the highest initial frequency of this genotype (0.63), while JC and HUN showed lower frequencies (0.40 and 0.13, respectively), suggesting that target-site mutations contributed differently to the observed resistance levels. These baseline gene expression profiles reflect inherited resistance mechanisms resulting from field exposure, particularly metabolic detoxification, and cuticular modifications, both well-established contributors to pyrethroid resistance in *Ae. aegypti*. Interestingly, MER also showed the fewest overexpressed resistance-related genes, with only 1 CP, 7 CYPs, and 2 GSTs. This suggests that its resistance profile is mainly linked to specific metabolic detoxification pathways and is characterized by already established kdr-mediated resistance [15]. In contrast, JC exhibited greater transcriptional activity, including 35 CPs, 9 CYPs, and 4 GSTs, indicating a combination of cuticular and metabolic resistance mechanisms. Although HUN had the lowest RR_50_, it showed the highest number of overexpressed genes, including 39 CPs, 15 CYPs, and 1 COE. This suggests a more diversified resistance profile, dominated by cuticular changes and supported by metabolic detoxification. The initially low kdr frequency in HUN may be compensated by other mechanisms [15]. Similar associations between kdr mutation fixation and transcriptomic changes under pyrethroid selection have been described in *An. gambiae* [61], and in *Ae. aegypti*, where expression of detoxification and cuticular genes played key roles even in non-kdr individuals [59]. This variability underscores the impact of local environmental factors and insecticide pressures on the selection of distinct resistance mechanisms within field populations [16,62,63,64].

Although numerous cuticular protein genes were overexpressed in the F_S0_ populations when compared to the susceptible strain, this pattern changed after insecticide selection, with most of these genes downregulated in F_S4_ groups relative to both F_S0_ and NOr. This suggests that the initial overexpression may primarily reflect adaptation to various environmental pressures rather than being directly linked to the resistance phenotype. Similar patterns were also observed in resistant *Ae. aegypti* from Puerto Rico, where most cuticular-related protein genes, particularly those associated with chitin and chitinase, were consistently downregulated following selection [41]. Additionally, cuticular thickening and increased polysaccharide content were detected in an *Ae. aegypti* strain with confirmed metabolic resistance to permethrin despite no detectable changes in cuticular hydrocarbons or phenolic biopolymers. These structural changes were hypothesized to arise from broader metabolic adjustments related to cytochrome P450 overexpression rather than being directly induced by insecticide exposure [65]. Overall, these findings highlight that cuticular gene expression and structural modifications are highly context-dependent and may reflect complex physiological responses to local environmental and metabolic conditions beyond insecticide pressure alone.

### 4.2. Transcriptomic Responses to Deltamethrin Selection in F_S4_ vs. F_S0_

Following four generations of deltamethrin selection, all three populations exhibited further reductions in susceptibility but varying degrees of transcriptomic change. HUN showed the greatest reduction (5.32-fold), followed by MER (1.44-fold) and JC (1.37-fold). In HUNF_S4_, overexpression of CYP6AG4 and CYP6M5 was observed. CYP6AG4 has been previously associated with resistance to permethrin in populations from Tapachula, Mexico [59] and was also reported to be overexpressed following selection with lambda-cyhalothrin [66]. Interestingly, a proteomic analysis found that CYP6AG4 protein was more abundant in the susceptible New Orleans strain than in resistant strains from French Guiana [57], suggesting that CYP6AG4 may reflect other physiological roles beyond its contribution to resistance. CYP6M5 has also been implicated in pyrethroid resistance in *Ae. aegypti* populations from the Cayman Islands [67] and to the organophosphate fenitrothion [68]. However, its expression was reported to be downregulated in a population selected with permethrin [53].

In JCF_S4_ selection-induced expression of CYP307A1, a Cytochrome P450 is involved in ecdysone synthesis and chitin regulation, which has been shown to play essential developmental roles in multiple insect species [69,70,71]. Its overexpression following deltamethrin selection may reflect physiological adaptations related to development and reproductive fitness rather than direct detoxification.

MERF_S4_ showed an increased expression of Ae-Aper50, a chitin-binding protein associated with the peritrophic matrix (PM), a physical barrier in the midgut involved in digestion and pathogen defense. This protein has been previously linked to midgut structural responses to blood feeding and metabolic stress in *Ae. aegypti* [72,73,74].

### 4.3. Shared Patterns and Functional Responses in F_S4_

Cytochrome P450 enzymes play a crucial role in metabolizing endogenous compounds and detoxifying insecticides and other xenobiotics [75]. Our transcriptomic data revealed the downregulation of multiple CYP450 genes in the F_S4_ groups. This pattern may reflect metabolic trade-offs resulting from continuous insecticide exposure or shifts in post-transcriptional regulation, similar to what has been observed in other resistant mosquito strains. A proteomic study conducted by Epelboin et al. [57], found that certain CYP proteins were less abundant in resistant strains compared to their susceptible counterparts. Moreover, many detoxification genes, including CYPs, have been found to be regulated by circadian rhythms in *An. gambiae*, suggesting that temporal factors may also influence expression patterns [76]. Previous studies have also reported reduced CYP expression following insecticide selection. For instance, several CYP genes were underexpressed in *Ae. aegypti* selected with permethrin [53], and downregulation was also observed after just one generation of exposure to the same insecticide [56]. More recently, six CYP genes were found to be downregulated in adults, but not in larvae, of a *Culex quinquefasciatus* strain selected with deltamethrin [77]. This further supports the idea that detoxification gene suppression may occur under insecticide pressure. Overall, these findings suggest that multiple factors, including developmental stage, insecticide exposure history, and potential compensatory physiological responses, can modulate CYP450 expression.

The downregulation of detoxification genes observed in FS4 may also reflect fitness trade-offs associated with maintaining resistance. Previous research using these same populations showed that deltamethrin selection led to reduced survival, fecundity, egg viability, and intrinsic growth rates, supporting the idea that resistance imposes physiological costs (Gonzalez-Santillan et al., 2022) [78].

CYP307A1/Spook (AAEL009762) is a regulatory cytochrome P450 gene involved in ecdysone synthesis, a key hormone for insect development and reproduction, as demonstrated in *Bombyx mori* larvae [69]. Similarly, its role in regulating chitin synthesis and reproductive fitness has been shown in *Spodoptera litura*, where CYP307A1 upregulation is co-regulated with the transcription factor Maf. The knockdown of CYP307A1 in *S. litura* resulted in a significant reduction in ecdysone production and chitin deposition, highlighting its critical developmental functions [70]. Interestingly, this same gene was not expressed in larval stages of *Drosophila* but was in follicular cells of the ovary from stage 8 of oogenesis, suggesting an important role in embryonic viability [71]. In this context, the overexpression of CYP307A1 following selection with deltamethrin, as observed in the biological comparisons of HUNF_S4_ vs. HUNF_S0_ and JCF_S4_ vs. JCF_S0_, may be linked to its essential role in development and embryonic viability. While its specific connection to insecticide resistance remains unclear, it is possible that its involvement in processes such as membrane synthesis or hormonal regulation indirectly contributes to physiological adaptations following insecticide exposure.

Our findings also identified overexpression of Ae-Aper50 (AAEL002467), a chitin-binding protein likely involved in the midgut peritrophic matrix (PM), which has been associated with mosquito response to blood feeding and digestion [51]. In adult mosquitoes, the PM is synthesized within hours of blood ingestion and serves as a semi-permeable layer composed of chitin fibrils, glycoproteins, and proteoglycans. This matrix protects the midgut from abrasive food particles, maintains structural integrity, and serves as a barrier against pathogen invasion, such as *Plasmodium gallinaceum* [79,80]. Several genes involved in PM synthesis in *Ae. aegypti* have been identified, including AeGfat-1, AeCs, and the peritrophins AAEL004798, AAEL006953, and Aper50. Among these, AeGfat-1 and Aper50 were significantly upregulated within 6 h post-blood meal, highlighting their critical roles in PM formation [72,73,74]. The overexpression of Ae-Aper50 in the biological comparison of MERF_S4_ vs. MERF_S0_ is likely associated with stress tolerance and synthesis of a thicker PM as a physical barrier. This supports the idea that internal physiological defenses, such as the PM, complement external cuticular modifications in enhancing deltamethrin resistance. Consistently resistant *Ae. aegypti* strains have shown structural changes in the cuticle, including thickening and increased polysaccharide content, possibly arising from metabolic adaptations [65]. Mucins, concentrated in the PM, resemble vertebrate mucosa in their protective role, reinforcing the significance of midgut structural defenses [81].

Following GOEA, the differentially expressed genes shared across the F_S4_ vs. F_S0_ included those potentially involved in detoxification, metabolic regulation, active transport, target-site modification, and adaptive gene control. Many of these genes encode proteins with catalytic and binding activities that may contribute to resistance. Genes involved in methyl group transfer and nucleic acid modification may participate in regulatory pathways modulating detoxification enzyme expression or function. Epigenetic mechanisms, such as RNA methylation and DNA methyltransferase activity, have been shown to regulate resistance genes and enable adaptive changes [82,83]. Additionally, genes linked to ATP hydrolysis are likely involved in energy-dependent detoxification, such as ABC transporter-mediated efflux [80]. Genes annotated with binding activity, including those potentially involved in insecticide sequestration, may help reduce the effective concentration of toxic compounds at their target sites. Binding proteins may reduce insecticide bioavailability at the point of contact, as demonstrated for SAP2 in *An. gambiae*, which binds pyrethroids at the point of contact, limiting their penetration and neurotoxic action [32].

Genes with RNA- and DNA-binding functions may also participate in post-transcriptional and transcriptional regulation of resistance-related pathways. Non-coding RNAs and their associated networks (e.g., lncRNA/circRNA–miRNA–mRNA) have been shown to regulate gene expression in response to insecticide exposure, modulating the expression of detoxification enzymes and contributing to resistance phenotypes [84]. Although SAP2 has not been functionally characterized in *Aedes* species, a recent transcriptomic study by Spadar et al. (2024) [85] identified SAP2 orthologs in *Ae. aegypti* but found no significant overexpression in deltamethrin-resistant populations, suggesting its role in resistance remains unclear.

Beyond metabolic and regulatory functions, several immune-related genes were upregulated in F_S4_. Notably, genes with helicase activity, including MOV-10, a known stress-response gene, were consistently overexpressed across all F_S4_ groups. This pattern may reflect the activation of immune and stress pathways following prolonged insecticide selection. Insecticides have been shown to modulate both cellular and humoral immune responses, which can enhance or suppress immunity depending on the compound and dose [86,87]. MOV-10 has been associated with antiviral defense and immune signaling in insects, particularly through RNA interference and post-transcriptional regulation, as shown in mosquito models [88]. The crosstalk between immune and detoxification pathways suggests resistance may involve a broader physiological response to mitigate xenobiotic stress.

Although this study focused on protein-coding genes due to annotation constraints, it is important to note that non-coding RNAs and pseudogenes may also play regulatory roles in the response to insecticide selection. Their exclusion represents a current limitation, and future studies incorporating functional analysis of lncRNAs, circRNAs, and pseudogene-derived transcripts may reveal additional regulatory mechanisms contributing to resistance in *Ae. aegypti*. In particular, validation of epigenetic pathways through methods such as bisulfite sequencing or methylation-specific PCR would help determine whether methylation processes are involved in the adaptive response to insecticide pressure.

Overall, the gene expression profiles observed in this study reflect both inherited and selection-induced resistance mechanisms in *Ae. aegypti*, resulting from field exposure and deltamethrin selection. These include well-established processes such as metabolic detoxification and cuticular remodeling, as well as less-characterized pathways involving stress response, immune activation, and midgut structural defenses.

## 5. Conclusions

This study highlights key transcriptomic changes associated with deltamethrin selection in *Ae. aegypti*. The differential expression of detoxification-related genes, cuticular proteins, and stress-response elements indicates that resistance involves multiple interacting mechanisms. The downregulation of cuticular protein genes in selected populations, compared to their baseline counterparts, suggests a role in initial tolerance rather than selection-driven resistance. In contrast, the consistent overexpression of genes such as CYP6AG4, CYP6M5, CYP307A1, and Ae-Aper50 supports the involvement of metabolic and midgut-related responses in the adaptation to insecticide pressure. The inclusion of immune and stress-associated genes further implies that deltamethrin selection may activate broader physiological pathways. These findings enhance our understanding of resistance dynamics and provide a basis for future studies exploring the functional roles and field relevance of these candidate genes.

## Figures and Tables

**Table 1:** Lethal concentrations (LC_50_) for *Ae. aegypti* females in the non-selected generation F_S0_ ^1^ and deltamethrin-selected generation F_S4_ ^1^.

Strain	Generation	N ^2^	LC_50_ (mg/Bottle)	CI 95% ^3^	Slope ± SE ^4^	RR_50_ ^6^
Merida	F_S0_	466	5.35 (4.73–6.56)	4.73–6.56	1.06 (0.09)	134
	F_S4_	501	7.73 (6.93–8.61)	6.93–8.61	1.92 (0.17)	193
Hunucma	F_S0_	424	0.25 (0.20–0.29)	0.20–0.29	1.24 (0.12)	6
	F_S4_	260	1.33 (1.16–1.52)	1.16–1.52	2.45 (0.26)	33
Jose Cardel	F_S0_	481	1.64 (1.46–1.83)	1.46–1.83	1.89 (0.18)	41
	F_S4_	420	2.26 (2.07–2.45)	2.07–2.45	2.81 (0.25)	57
New Orleans ^5^	-	492	0.04 (0.03–0.05)	0.03–0.05	1.11 (0.10)	1

^1^ From Contreras-Perera et al. [15]. ^2^ N: sample size. ^3^ 95% confidence intervals. ^4^ Slope of the regression line Probit-log, the standard error in parentheses. ^5^ New Orleans: susceptible reference strain. ^6^ RR_50_: resistance ratio LC_50_ of the non-selected generation (F_S0_)/LC_50_ susceptible strain (NOr).

**Table 2:** Number of differentially expressed genes in comparisons between selected (F_S4_) and non-selected (F_S0_) generations following deltamethrin selection in different *Aedes aegypti* populations.

Biological Comparisons	# of Genes Tested	DE Genes (log_2_FC > 1, FDR ≤ 0.05)		DE Genes (log_2_FC > 1, FDR ≤ 0.01)	
		Up	Down	Up	Down
HUNF_S0_ vs. NOr	10,210	1270	979	973	631
HUNF_s4_ vs. HUNF_S0_	9714	407	935	181	668
HUNF_S4_ vs. NOr	10,054	944	789	816	477
JCF_S0_ vs. NOr	10,188	1400	860	1247	648
JCF_S4_ vs. JCF_S0_	9605	497	1198	326	909
JCF_S4_ vs. NOr	10,096	1723	1677	1555	1291
MERF_S0_ vs. NOr	9840	346	348	224	201
MERF_S4_ vs. MERF_S0_	6672	451	880	311	610
MERF_S4_ vs. NOr	9830	650	947	387	497

Comparisons include populations from Jose Cardel (JC), Merida (MER), Hunucma (HUN), and the susceptible strain New Orleans (NOr). F_S0_: baseline generation, non-selected; F_S4_: after four generations of selection with deltamethrin. DE: differentially expressed; log_2_FC: log base 2 of the fold change in gene expression between conditions (log_2_FC > 1 corresponds to FC > 2); FDR: false discovery rate.

**Table 3:** Significantly overexpressed genes after deltamethrin selection (F_S4_ vs. F_S0_) in three *Aedes aegypti* populations (FDR ≤ 0.01 and |log_2_FC| > 1).

Gene ID	Description	log_2_FC	FDR	Group
HUNF_S4_ vs. HUNF_S0_				
AAEL007010	CYP6AG4	2.86	5.93 × 10^−6^	CYP
AAEL009117	CYP6M5	1.52	8.47 × 10^−4^	CYP
AAEL009762	CYP307A1	2.06	0.0126	CYP
JCF_S4_ vs. JCF_S0_				
AAEL009762	CYP307A1	3.53	1.76 × 10^−5^	CYP
MERF_S4_ vs. MERF_S0_				
AAEL002467	Ae-Aper50	5.52	3.29 × 10^−5^	CP

Hunucma (HUN), Jose Cardel (JC), Merida (MER); F_S0_, baseline generation (non-selected); F_S4_, four generations of selection with deltamethrin. Abbreviations of the functional groups: cytochrome P450s (CYPs), cuticular proteins (CPs).

## Data Availability

Sequence data generated by this study are available at the Sequence Read Archive (SRA) BioProject PRJNA1259471. All other relevant data and scripts for this work are available from the corresponding author upon request.

## Supplementary Materials

The following supporting information can be downloaded at: https://www.mdpi.com/article/10.3390/tropicalmed10060171/s1, File S1: Bioinformatics pipeline used for RNA-seq quality filtering, alignment, and read quantification. File S2: Bioinformatics pipeline and differential gene expression analysis for deltamethrin selection in *Aedes aegypti*. File S3: Full results of differential gene expression analysis. All the genes having a transcription signal are mapped with their gene expression count, FDR, fold change expression, and functional annotations. File S4: The DGEs that overlapped multiple pairwise comparisons for the different experiments. The overlapped DGEs were mapped to their logFC expression, functional description, and gene ontology. File S5: Functional description and GO annotation of the protein-coding genes from VectorBase and Blast2GO. File S6: GO enrichment analysis of DE genes (up and downregulated) F_S4_ vs. F_S0_ comparisons. Table S1: Summary statistics of the RNA-seq reads: This table shows the key statistics describing the RNA-seq reads before and after quality filtering, the alignment of the filtered reads to the reference genome and the read quantification results. Figure S1: Gene ontology enrichment analysis (GOEA) of differentially expressed genes from F_S4_ vs. F_S0_ comparisons.
