# Supplementary material for: Deltamethrin Selection Drives Transcriptomic Changes in Detoxification, Immune, and Cuticle Genes in Aedes aegypti
Source: Trop Med Infect Dis. 2025 Jun 17;10(6):171. doi: 10.3390/tropicalmed10060171 (PMC12197768; doi:10.3390/tropicalmed10060171)
Supplement: Supplementary file 1 [file tropicalmed-10-00171-s001.zip › Figure S1.pdf]

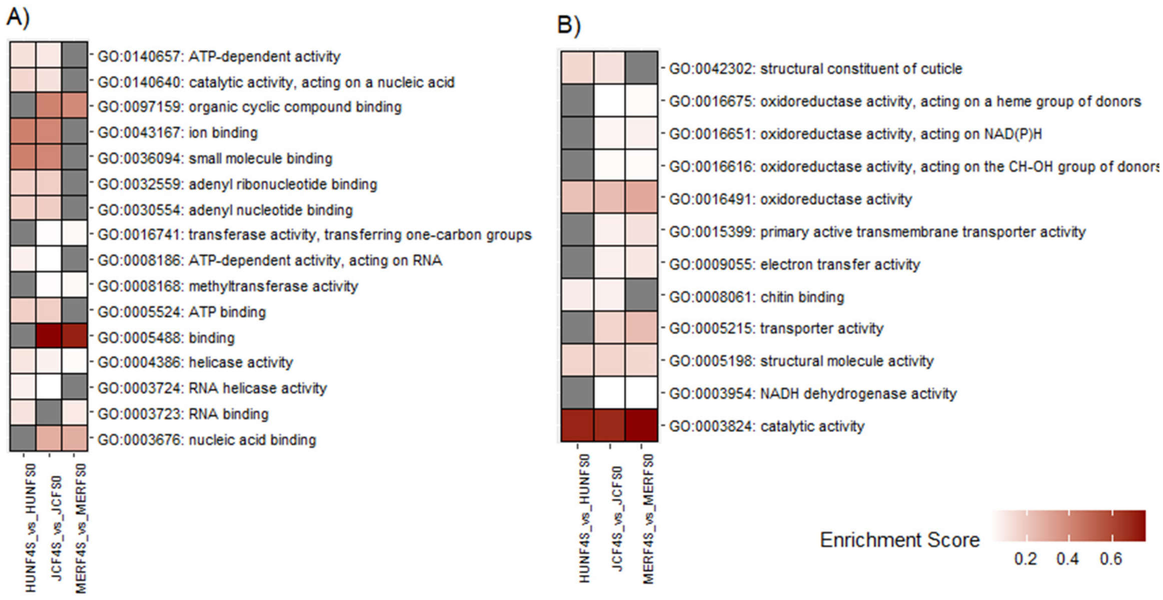

Figure S1. Gene Ontology enrichment analysis (GOEA) of differentially expressed genes from F_{S4} vs. F_{S0} comparisons. (A) GOEA of upregulated genes. (B) GOEA of downregulated genes. Only GO terms that were significantly enriched in at least two comparisons and had an enrichment score ≥ 0.05 are shown. The enrichment score represents the fraction of study genes (up- or downregulated) associated with each molecular function GO term.
